# Effect of Virtual Reality on Stress Reduction and Change of Physiological Parameters Including Heart Rate Variability in People With High Stress: An Open Randomized Crossover Trial

**DOI:** 10.3389/fpsyt.2021.614539

**Published:** 2021-08-10

**Authors:** Hyewon Kim, Dong Jun Kim, Seonwoo Kim, Won Ho Chung, Kyung-Ah Park, James D. K. Kim, Dowan Kim, Min Ji Kim, Kiwon Kim, Hong Jin Jeon

**Affiliations:** ^1^Department of Psychiatry, Hanyang University Medical Center, Seoul, South Korea; ^2^Department of Psychiatry, Depression Center, Samsung Medical Center, Sungkyunkwan University School of Medicine, Seoul, South Korea; ^3^Department of Health Sciences and Technology, Department of Medical Device Management and Research, and Department of Clinical Research Design and Evaluation, Samsung Advanced Institute for Health Sciences and Technology (SAIHST), Sungkyunkwan University, Seoul, South Korea; ^4^Statistics and Data Center, Research Institute for Future Medicine, Samsung Medical Center, Seoul, South Korea; ^5^Department of Otorhinolaryngology, Samsung Medical Center, Sungkyunkwan University School of Medicine, Seoul, South Korea; ^6^Department of Ophthalmology, Samsung Medical Center, Sungkyunkwan University School of Medicine, Seoul, South Korea; ^7^AR Lab, Samsung Research, Samsung Electronics Co., Ltd, Seoul, South Korea; ^8^Advanced Solution Team, Samsung Research, Samsung Electronics Co., Ltd, Seoul, South Korea; ^9^Department of Psychiatry, Kangdong Sacred Heart Hospital, Hallym University College of Medicine, Seoul, South Korea

**Keywords:** virtual reality, biofeedback, heart rate variability, stres, stress reduction

## Abstract

**Introduction:** Although, attempts to apply virtual reality (VR) in mental healthcare are rapidly increasing, it is still unclear whether VR relaxation can reduce stress more than conventional biofeedback.

**Methods:** Participants consisted of 83 healthy adult volunteers with high stress, which was defined as a score of 20 or more on the Perceived Stress Scale-10 (PSS-10). This study used an open, randomized, crossover design with baseline, stress, and relaxation phases. During the stress phase, participants experienced an intentionally generated shaking VR and serial-7 subtraction. For the relaxation phase, participants underwent a randomly assigned relaxation session on day 1 among VR relaxation and biofeedack, and the other type of relaxation session was applied on day 2. We compared the State-Trait Anxiety Inventory-X1 (STAI-X1), STAI-X2, the Numeric Rating Scale (NRS), and physiological parameters including heart rate variability (HRV) indexes in the stress and relaxation phases.

**Results:** A total of 74 participants were included in the analyses. The median age of participants was 39 years, STAI-X1 was 47.27 (SD = 9.92), and NRS was 55.51 (SD = 24.48) at baseline. VR and biofeedback significantly decreased STAI-X1 and NRS from the stress phase to the relaxation phase, while the difference of effect between VR and biofeedback was not significant. However, there was a significant difference in electromyography, LF/HF ratio, LF total, and NN50 between VR relaxation and biofeedback.

**Conclusion:** VR relaxation was effective in reducing subjectively reported stress in individuals with high stress.

## Introduction

The stress-vulnerability model proposes that, depending on the intensity of the elicited stress and the threshold for tolerating it, i.e., one's vulnerability, a stressful crisis may be contained homeostatically or may lead to psychiatric disorders (1). This model has been useful for identifying and managing psychiatric disorders, and many studies have examined how stress affects the brain and what traits of individuals affect vulnerability. The hypothalamo-pituitary-adrenal (HPA) axis has been suggested as being associated with the development of major psychiatric disorders, such as depression, mania, psychosis, and anxiety disorders (2–4). Stress increases the level of cortisol, and persistent hypercortisolemia leads to glucocorticoid receptor tolerance (5). This change also affects the hippocampus, a brain region rich in corticosteroid receptors (6). Hippocampal dysfunction can lead to inappropriate emotional responses (7), and changes of hippocampal volume have been reported in psychiatric disorders, including schizophrenia, post-traumatic stress disorder, borderline personality disorder, and depression (8–11).

Personality traits are known to explain an individual's responsiveness to stress and vulnerability (12–14). The factor of neuroticism was confirmed to have a relationship with stress-related psychopathologies in many studies (15–18), and in particular, high anxiety is known to be a critical risk factor of hyper-responsiveness to stress and of vulnerability to developing the psychopathologies of anxiety disorders and depression (19, 20). Considering these factors, proper management of stress in the vulnerable group with high anxiety will be important for preventing the development of psychiatric disorders.

Since the introduction of virtual reality (VR) in the 1950's, equipment related to VR systems has been gradually upgraded and made lightweight, and VR has been actively used in various fields. In the medical field, attempts have been made to use VR for diagnosis and treatment of disease. The use of VR in psychiatry seems to have an advantage in effectively educating and training patients to deal with negative emotions, such as anxiety. In a variety of studies, VR was applied to the treatment of psychiatric diseases. Since clinical studies using VR in treatment of acrophobia in the 1990's (21), randomized controlled studies have been conducted not only in patients with psychiatric disorders, including post-traumatic stress disorder (22–25), anxiety disorders (26), phobias (27, 28), psychotic disorders (29–31), and cognitive disorders (32), but also in people with anxiety (33, 34) or physical pain (33, 35, 36), and have demonstrated the effects of VR on reduction of symptoms and improved management of diseases.

There have been several studies investigating the effect of VR on stress reduction. A study using a mobile application that delivers VR showed that stress level was reduced in a cost-effective and accessible manner by VR (37). Another VR study showed objective and subjective effects of relaxation by means of VR with natural scenes compared to scenes with indoor settings (38). In a work population with high stress, when immersive natural scenarios were applied to learning specific relaxation techniques, there was a reduction in chronic trait anxiety and an increase in coping skills (39). A study tried mindfulness using VR and showed the possibility of mindful attention and relaxation by means of VR (40).

Biofeedback provides non-invasive, effective psychophysiological intervention for various psychiatric disorders and is widely used in clinical settings (41). According to a meta-analysis, biofeedback training was effective in reducing self-reported stress and anxiety (42), and such a result was repeated in healthy people with high stress (43). A previous study tried to compare the relaxing effect of VR and biofeedback among healthy participants, where four relaxing treatments were made by combining display type (VR vs. computer screen) and biofeedback (electrodermal activity biofeedback vs. no biofeedback) and applied randomly to subjects, but there was no treatment-specific difference in subjective stress or physiological arousal (44).

As the Research Domain Criteria (RDoC) was developed by the National Institute of Mental Health, the need for alternatives to the traditional psychiatric nosology, such as a dimensional system in which classification is derived inductively, has emerged. As important transdiagnostic biomarkers, physiologic parameters can be used to measure physical responses to induced stress. Stress increases one's arousal and leads to various bodily responses, such as an accelerated heart rate, pupil dilatation, increased galvanic skin response, increased finger-pulse volume, and increased electromyography (EMG) activity (45). In addition, although fluctuation in the beat-by-beat heart period is an intrinsic characteristic of cardiac functioning, heart rate variability (HRV) represents the ability of the heart to respond to physiological and environmental stimuli (46). Previous studies have shown the possibility of HRV as a psychophysiological measurement for reactivity to stress (47–50). For example, increased stress was associated with decreased inter-beat interval (IBI) and increased the low-frequency band/the high-frequency band ratio (LF/HF ratio) (51), and anxiety was associated with decreased root mean square of the successive differences (RMSSD) (52).

In this study we aimed to identify the stress-reduction effect of VR relaxation compared to that from biofeedback after exposure to stress in people with high stress, as measured by psychological scales and physiological parameters, including HRV indexes. We tried to examine the following hypotheses in this study:

1. After exposure to stress, the effect of stress reduction in a high-stress group will be greater in a relaxation session by VR than in one by biofeedback.
2. Change of stress after a relaxation session will be measurable by means of physiological parameters, including HRV indexes.

## Methods

### Participants

We recruited 83 healthy adult volunteers with high stress, age 19 or more, from October 2016 to January 2018. We defined high stress as a score of 20 or more on the Perceived Stress Scale-10 (PSS-10) (53). Inclusion criteria were healthy persons who voluntarily participated in this study and who had no problem in understanding the study procedures and controlling the VR equipment. Those who had major psychiatric disorders, suicidal risk, neurological illnesses, including stroke or epilepsy, or serious medical illnesses were excluded. In addition, those who had medical or surgical history of psychiatric, otologic, or ophthalmologic disorders or problems with neck movements were also excluded. All participants were drug-naïve when a sample measurement was done at the baseline evaluation. At the baseline screening visit, participants were evaluated by a psychiatrist (HJJ). A psychologist who specialized in this psychiatric evaluation administered the Korean version of the Mini International Neuropsychiatric Interviews (MINI) (54) according to the Diagnostic and Statistical Manual of Mental Disorders (DSM-5) (55) to the subjects to evaluate psychiatric disorders. Our study was approved by the Institutional Review Board of the Samsung Medical Center (IRB No. SMC 2016-10-007-004), and all participants gave written informed consent at enrollment in the study.

### Study Procedure

#### Study Design

**Figure 1:**
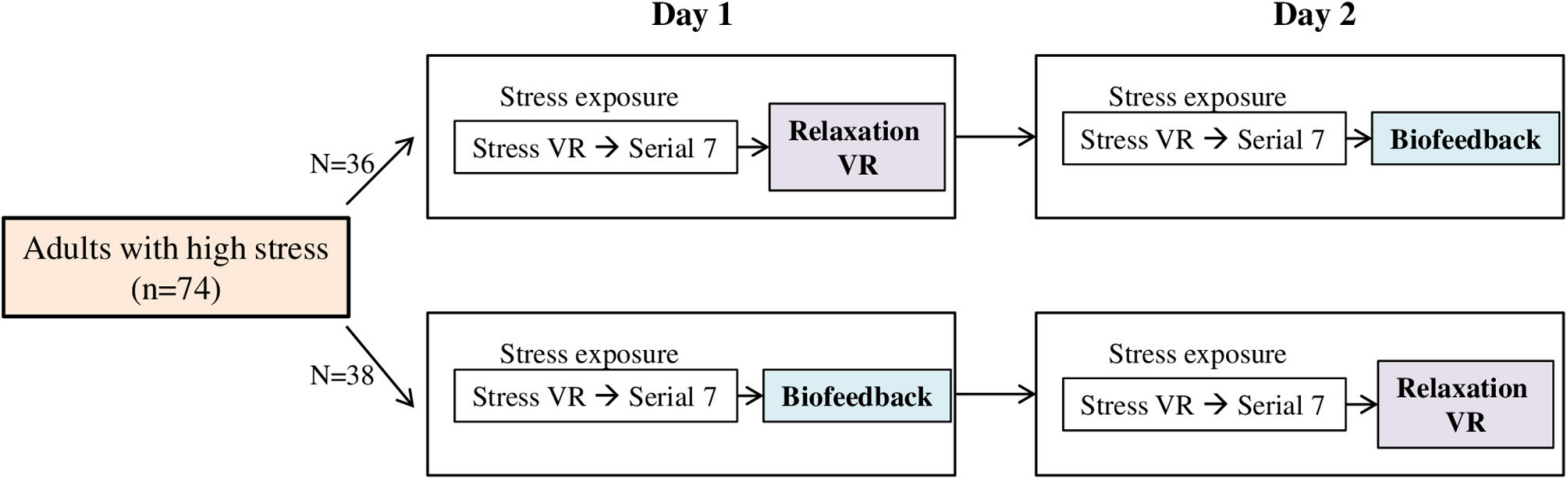
Study design. VR, virtual reality.

**Figure 2:**
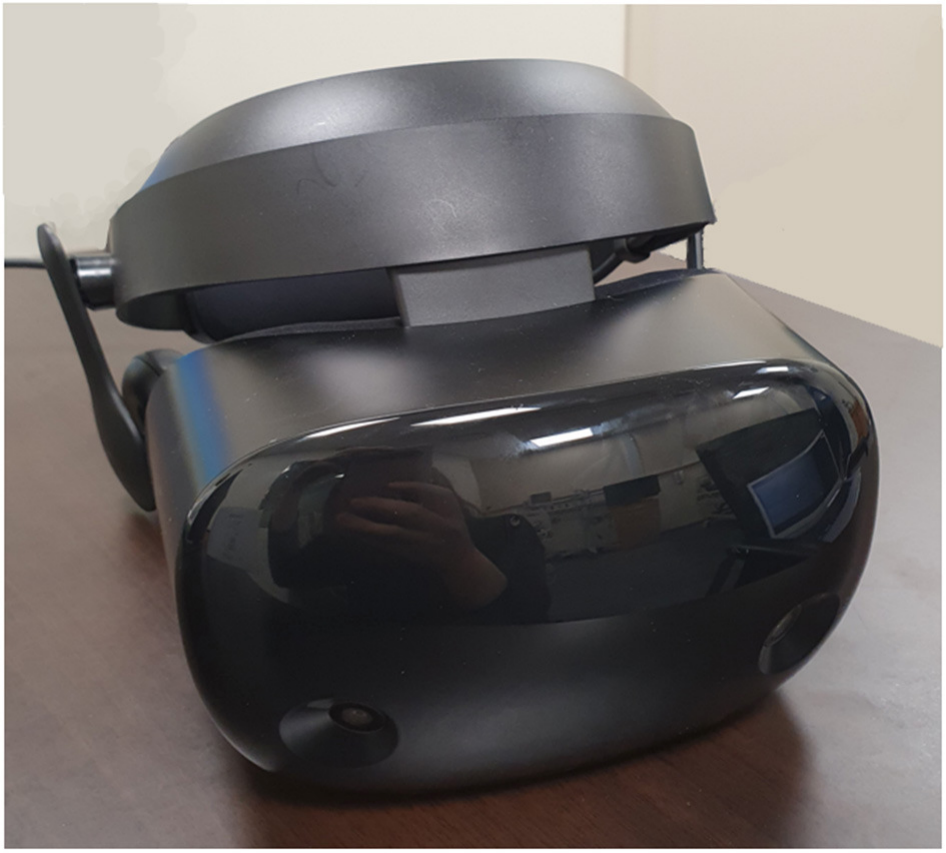
VR device. VR, virtual reality.

**Figure 3:**
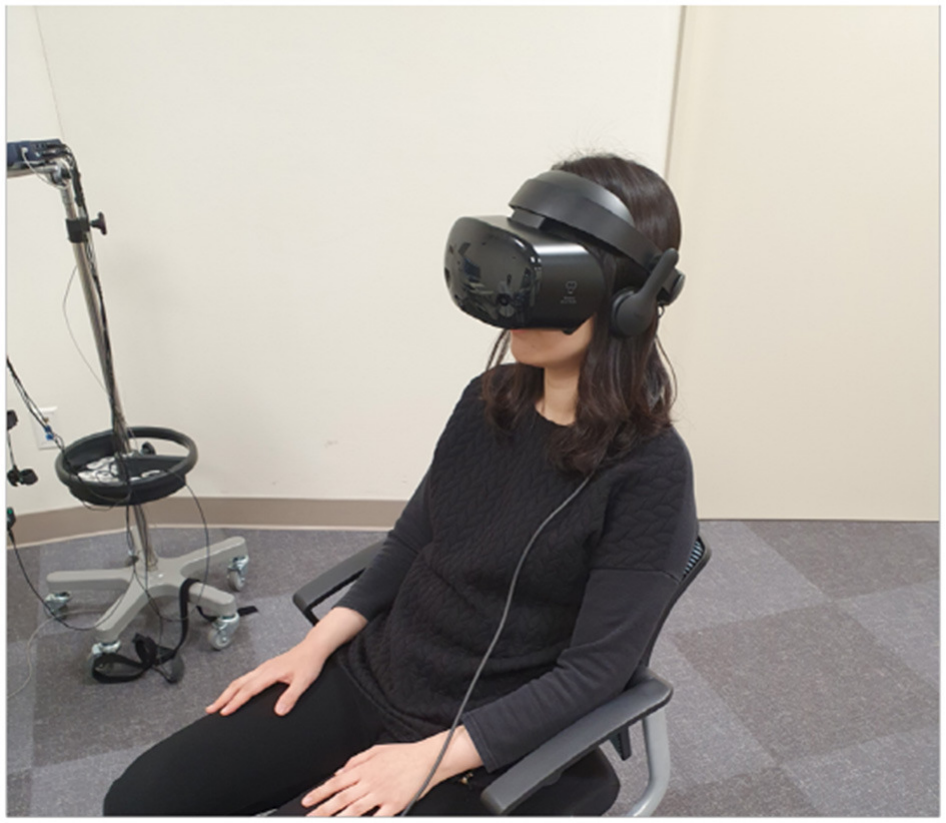
Snapshot of VR relaxation session. VR, virtual reality.

This study used an open, randomized, two-period, two-treatment crossover design. The study process consisted of a baseline phase, stress phase, and relaxation phase, and was aimed to compare the differences of subjective stress reduction and physiological parameters when VR or biofeedback was applied in the relaxation phase. On day 1, participants underwent a randomly assigned relaxation session. On day 2, the same process was conducted in the stress phase, and the other type of relaxation session was applied in the relaxation phase. We compared the differences in stress reduction and physiological parameters according to the type of relaxation sessions for participants (Figure 1). All research was conducted in a room that was exclusively prepared to block outside noise in the Clinical Trial Center located in Samsung Medical Center. Samsung Gear VR (Samsung Electronics Co., Ltd., Suwon, South Korea) was used in the stress-exposure phase and relaxation phase, and the head-mounted display (HMD) device included separate screens for each eye, integrated head tracking, and stereo earphones (Figures 2, 3).

#### Stress Exposure

In order to apply stress in a limited laboratory environment during a short time, we operationally set a protocol to induce stress, in which dizziness and discomfort were induced by experiencing VR videos with a high degree of movement, and cognitive load was given by serial subtraction. Symptoms of motion sickness such as dizziness and discomfort caused by visual-vestibular mismatch elicited stress-related physiological changes including activation of the HPA axis and elevation in level of cortisol (56, 57). In addition, a previous study suggested serial subtraction as an effective task in inducing a stress response as the cerebral blood flow changed and the level of salivary cortisol increased to reach a peak after the end of the task (58). First, participants experienced an intentionally generated shaking VR. The original video was provided by the Korea Land and Geospatial Informatix Corporation. The video was artificially modified for this study by adding a roll swing of a sine waveform of 30 Hz in the *z*-axis direction with 0.008°/s for each grade and image movements of 0.3 and 0.38°/s. The quantitative degree of movement of the VR video was set by referring to previous studies to sufficiently induce dizziness and discomfort in the subjects (59, 60). Participants were exposed to a VR video that walked on a shaky path for 3 min and 30 s, and after a break of 3 min and 30 s, they were exposed to a VR video once again that differed in the intensity of shaking from the first video. During the exposure to stress VR video, participants were asked to count the number of persons who appeared in the video in order to increase their attention to the video. After exposure to the stress VR video, a break of 3 min and 30 s was taken, after which the participants were asked to perform serial-7 subtraction for another 3 min and 30 s in order to be cognitively loaded.

#### Relaxation Sessions

Following the stress phase, we carried out relaxation sessions. On day 1, participants were exposed to VR relaxation or biofeedback as randomly assigned. On day 2, after the same process of stress exposure, the other type of relaxation session was applied as a crossover design. Both relaxation sessions using VR or biofeedback lasted for 10 min and 30 s.

**Figure 4:**
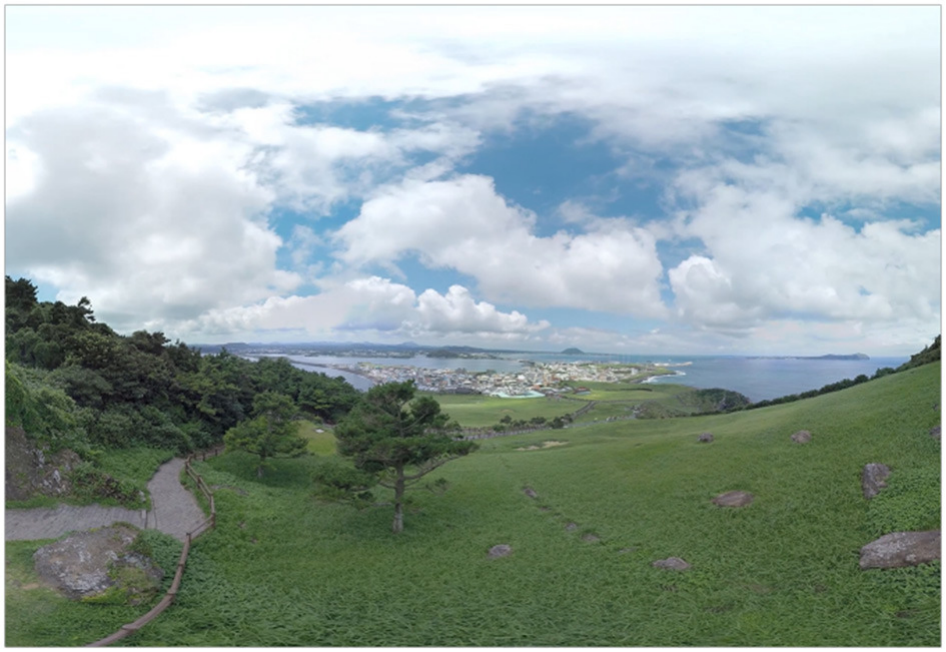
Immersive VR video. VR, virtual reality.

For the relaxation session using VR, the same device used in the stress phase, Samsung Gear VR (Samsung Electronics Co., Ltd., Suwon, South Korea), was used. During the session, participants were shown a VR video of immersive natural scenes while walking on a trekking course with famous scenery and with a relaxing soundtrack (Figure 4).

**Figure 5:**
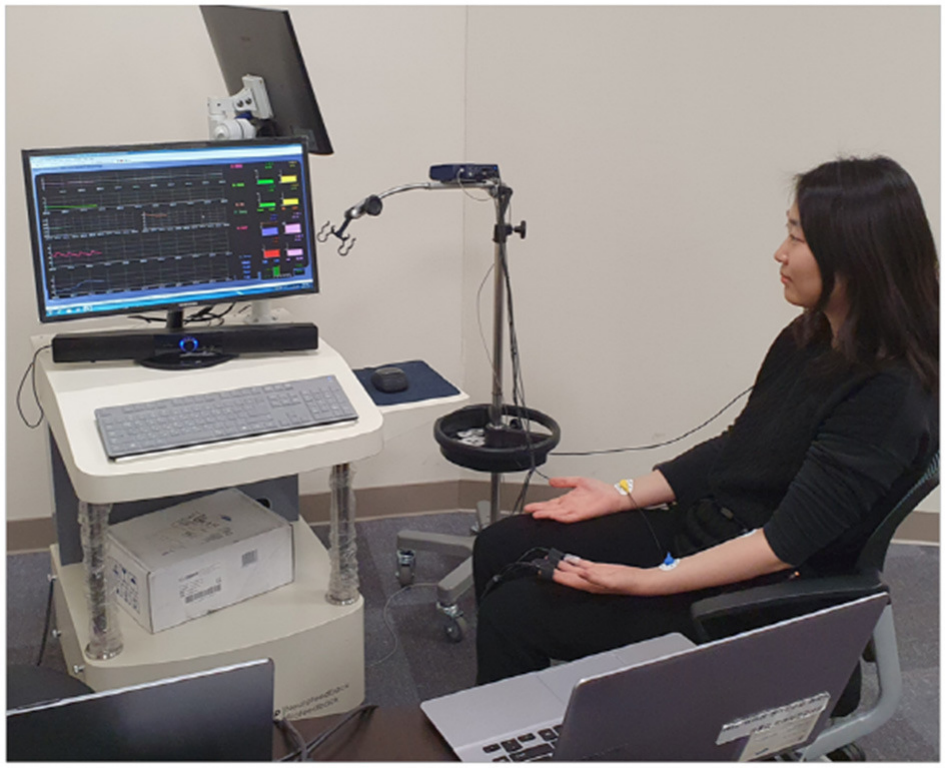
Snapshot of biofeedback session.

For the relaxation session using biofeedback, a computerized biofeedback system, ProComp Infiniti (Thought Technology, Ltd., Montreal, Canada), was used. We acquired information related to EMG, skin conductance, skin temperature, respiration, and heart rate/blood vessel pressure (HR/BVP) through sensors attached to the subject's body. Before the session, subjects learned relaxation reactions such as the decrease in EMG and skin conductance and increase in temperature, and were instructed to induce relaxation while watching these signals displayed on the screen during the session. In addition, the experimenter gave feedback to the participants when the parameters increased or decreased by 10% (Figure 5).

#### Psychological Evaluation

At baseline, participants were evaluated by the Korean version of psychological scales including the State-Trait Anxiety Inventory-X1 (STAI-X1), STAI-X2 (61), 0–100 Numeric Rating Scale (NRS) (62), Positive Affect and Negative Affect Schedule (PANAS) (63), Sheehan's Disability Scale (SDS) (64), and the five-level version of the EQ-5D (EQ-5D-5L) (65). STAI-X1 and NRS were measured repeatedly in each phase.

#### Acquisition of Physiological Parameters

Throughout the entire study, we acquired physiological parameters by means of sensors attached to the subject's body using the biofeedback device, ProComp Infiniti (Thought Technology, Ltd., Montreal, Canada). In addition to biofeedback parameters, HRV parameters, such as HR from inter-beat interval (IBI), mean power of the very-low-frequency band (VLF: 0.0033–0.04 Hz), mean power of the low-frequency band (LF: 0.04–0.15 Hz), mean power of the high-frequency band (HF: 0.15–0.4 Hz), ratio of LF to HF power (LF/HF ratio), number of interval differences of successive normal-to-normal (NN) intervals greater than 50 ms (NN50), percentage of NN50 (pNN50) standard deviation of NN (SDNN), and RMSSD were acquired. We included parameters extracted by monitoring for 3 min and 30 s after each phase in the analyses.

#### Simulator Sickness Questionnaire

We repeatedly measured the simulator sickness questionnaire (SSQ) (66) before and after the experience of intentionally generated shaking VR during the stress exposure session to evaluate sickness symptoms that may occur. SSQ consists of three subscales (nausea, oculomotor, and orientation), and a higher score means more severe symptoms.

### Outcomes

As the primary outcomes, we examined the change of anxiety or discomfort from the stress phase to the relaxation phase by means of psychological scales and physiological parameters, and compared the relaxation effect of VR and biofeedback.

There are individual differences in adaptation to VR equipment and satisfaction with immersion, presence, and interactivity experienced in a virtual environment (67). Moreover, sickness symptoms that may occur during VR applications can be an obstacle for relaxation through VR (68). Therefore, we defined responders as those who reported decreased NRS after VR relaxation and biofeedback compared to each stress exposure session. Among them, we compared the changes of physiological parameters during a VR session with those during a biofeedback session. In addition, we investigated the changes of SSQ before and after VR application in both responders and non-responders.

### Statistical Analyses

We examined the distribution of demographic and clinical characteristics of participants. In each phase, we examined the distributions of STAI-X1, NRS, and physiological parameters. We used paired *t-*tests and Wilcoxon signed-rank tests to compare the differences of scales and physiological parameters between the stress phase and the relaxation phase according to the type of relaxation session. All statistical analyses were done with SAS version 9.4 (SAS Institute, Cary, NC, USA).

## Results

### Baseline Demographic and Clinical Characteristics of Participants

Table 1 shows the demographic characteristics and baseline psychological evaluation data of the participants. Of the 83 enrolled subjects, two were lost to follow-up, seven were excluded from analysis because of data-extraction errors, and a total of 74 were included in the analysis. The median age of the participants was 39 years, with 37 men and women each. The mean baseline of STAI-X1 was 47.27 (SD = 9.92), and the mean baseline NRS was 55.51 (SD = 24.48).

**Table 1:** Baseline demographic and clinical characteristics of participants (*n* = 74).

**Demographic characteristics**	**No or median (range)**
Age (years)	39 (19–59)
Sex (male/female)	37/37
Education (years)	16 (6–22)
BMI (kg/m^2^)	23 (17–33)
Smokers/non-smokers	57/17
Alcohol consumption (bottles/week)	1.0 (0–9)
**Clinical characteristics**	**Mean (SD)**
STAI-X1	47.27 (9.92)
NRS	55.51 (24.48)
PSS-10	26.09 (4.64)
PANAS	22.26 (9.82)
SDS	15.66 (6.59)
STAI-X2	48.16 (9.88)
EQ-5D-5L	6.47 (1.63)

*BMI, Body mass index; STAI, State-Trait Anxiety Inventory; NRS, Numeric Rating Scale; PSS-10, Perceived Stress Scale; PANAS, Positive and Negative Affect Schedule; SDS, Sheehan Disability Scale; EQ-5D-5L, Five-level version of EQ-5D*.

### Changes of STAI-X1 and NRS According to the Type of Relaxation Session

Table 2 shows STAI-X1 and NRS at the baseline, stress, and relaxation phases when the relaxation session was applied by VR or biofeedback. The higher score on STAI-X1 indicated the more anxious state of a subject. When relaxation was induced with VR, it increased slightly from baseline 43.93 (SD = 10.05) to 45.22 (SD = 12.27) after stress exposure and decreased to 38.80 (SD = 9.96) after relaxation. When relaxation was induced by biofeedback, from baseline 44.76 (SD = 9.65), a similar level was maintained at 44.65 (SD = 11.67) after stress exposure and decreased to 38.80 (SD = 8.94) after relaxation. The difference in STAI-X1 from the stress phase to the relaxation phase was slightly higher for VR relaxation (M = 6.42, SD = 10.03) than for biofeedback (M = 5.85, SD = 7.51), but the difference between VR and biofeedback was not statistically significant (M = 0.57, SD = 9.58, *p* = 0.394) (Supplementary Figure 1).

**Table 2:** STAI-X1 and NRS for the study procedure according to the type of relaxation session (*n* = 74).

	**VR**	**Biofeedback**	***p*^a^**
	**Mean (SD)**		
**STAI-X1**			
Baseline	43.93 (10.05)	44.76 (9.65)	
Stress exposure	45.22 (12.27)	44.65 (11.67)	
Relaxation session	38.80 (9.96)	38.80 (8.94)	
Δ(Stress exposure – relaxation session)	6.42 (10.03)^***^	5.85 (7.51)^***^	0.394
**NRS**			
Baseline	47.36 (26.13)	49.77 (24.76)	
Stress exposure	53.80 (24.53)	55.62 (24.64)	
Relaxation session	41.43 (23.74)	42.45 (24.39)	
Δ(Stress exposure – relaxation session)	12.36 (20.04)^***^	13.18 (18.00)^***^	0.561

*STAI, State-Trait Anxiety Inventory; NRS, Numeric Rating Scale; VR, virtual reality*.

a *Wilcoxon signed-rank test*.

*** *p < 0.001*.

The higher score on the NRS indicated greater discomfort. When relaxation was induced with VR, it increased from baseline 47.36 (SD = 26.13) to 53.80 (SD = 24.53) at the stress phase and decreased to 41.43 (SD = 23.74) after the relaxation session. When relaxation was induced by biofeedback, NRS increased from baseline 49.77 (SD = 24.76) to 55.62 (SD = 24.64) after the stress phase and decreased to 42.45 (SD = 24.39) after the relaxation session. The difference in NRS from the stress phase to the relaxation phase was lower for VR relaxation (M = 12.36, SD = 20.04) than for biofeedback (M = 13.18, SD = 18.00), but the difference was not statistically significant (M = −0.81, SD = 18.95, *p* = 0.561) (Supplementary Figure 2).

### Changes of Physiological Parameters During the Baseline, Stress Phase, and Relaxation Phase

Table 3 compares the differences of physiological parameters from the stress phase to the relaxation phase according to the type of relaxation session. EMG decreased by 0.84 (SD = 4.53) in VR relaxation and decreased by 4.41 (SD = 20.60) in biofeedback, showing more decrease in biofeedback (*p* = 0.016). LF total decreased by 16.87 (SD = 119.59) in VR relaxation and increased by 38.57 (SD = 266.58) in biofeedback (*p* = 0.045). LF/HF decreased by 0.32 (SD = 2.43) in VR relaxation and increased by 2.66 (SD = 11.93) in biofeedback (*p* = 0.022). NN50 increased by 69.81 (SD = 74.66) in VR relaxation and increased by 50.74 (SD = 53.96) in biofeedback (*p* = 0.021) (Supplementary Table 1 and Supplementary Table 2).

**Table 3:** Changes of physiological parameters from the stress phase to the relaxation phase according to type of relaxation session (*n* = 74).

	**VR**	**Biofeedback**	***p*^a^**
	**Mean (SD)**		
ΔEMG	0.84 (4.53)^**^	4.41 (20.60)^***^	0.016
ΔSkin conductance	0.43 (0.65)^***^	0.51 (0.72)^***^	0.194
ΔTemperature	−0.82 (1.12)^***^	−0.80 (1.04)^***^	0.862
ΔRespiratory amplitude	0.33 (2.19)	−0.15 (2.69)	0.345
ΔHR/BVP	0.05 (0.08)^***^	0.07 (0.11)^***^	0.183
ΔHR from IBI	6.61 (4.83)^***^	5.65 (4.19)^***^	0.142
ΔVLF total	−2.87 (70.07)	−20.60 (78.26)^*^	0.091
ΔLF total	16.87 (119.59)^*^	−38.57 (266.58)	0.045
ΔHF total	68.73 (148.40)^**^	64.48 (192.99)	0.555
ΔHRV total	113.25 (290.96)^**^	33.42 (485.16)	0.054
ΔLF/HF	0.32 (2.43)	−2.66 (11.93)	0.022
ΔEKG IBI	−80.37 (63.90)^***^	−74.46 (65.41)^***^	0.140
ΔNN50	−69.81 (74.66)^***^	−50.74 (53.96)^***^	0.021
ΔpNN50	−7.09 (7.67)^***^	−5.24 (5.61)^***^	0.017
ΔSDNN	16.83 (60.24)	21.41 (50.67)^**^	0.595
ΔRMSSD	15.09 (59.58)	20.94 (50.73)^**^	0.892

*VR, virtual reality; EMG, electromyography; HR/BVP, heart rate/blood vessel pressure; IBI, inter-beat interval; VLF, very-low-frequency band; LF, low-frequency band; HF, high-frequency band; NN50, number of interval differences of successive normal-to-normal (NN) intervals >50 ms; pNN50, percentage of NN50; SDNN, standard deviation of NN; RMSSD, the root mean square of the successive differences*.

a *The two groups were compared with the paired t-test when the normality assumption was satisfied and the Wilcoxon signed-rank test if not*.

* *p < 0.05*,

** *p < 0.01*,

*** *p < 0.001*.

### Changes of Physiological Parameters According to the Type of Relaxation Session in the Responders

We defined the responders as the subjects with decreased NRS after VR relaxation and biofeedback compared to each stress exposure session and identified the changes in their physiological parameters before and after relaxation sessions. Among a total of 39 responders, EMG showed an average decrease of 0.80 (SD = 4.62) in VR relaxation, and an average decrease of 6.26 (SD = 27.04) in biofeedback (M = −5.46, SD = 27.37, *p* = 0.044). NN50 increased by 81.15 (SD = 84.63) in VR relaxation and increased by 52.28 (SD = 53.59) in biofeedback (M = −28.87, SD = 72.94, *p* = 0.020) (Table 4).

**Table 4:** Changes of physiological parameters from the stress phase to the relaxation phase according to the type of relaxation session in the responders^a^ (*n* = 39).

	**VR**	**Biofeedback**	***p*^b^**
ΔEMG	0.80 (4.62)^*^	6.26 (27.04)^***^	0.044
ΔSkin conductance	0.47 (0.68)^***^	0.58 (0.88)^***^	0.178
ΔTemperature	−0.91 (1.01)^***^	−0.95 (1.05)^***^	0.869
ΔRespiratory amplitude	0.27 (2.37)	−0.08 (2.04)	0.330
ΔHR/BVP	0.04 (0.06)^***^	0.07 (0.13)^***^	0.153
ΔHR from IBI	7.00 (5.45)^***^	5.83 (4.96)^***^	0.379
ΔVLF total	−12.06 (69.55)	−26.85 (89.59)	0.359
ΔLF total	−6.80 (115.16)	−83.44 (332.79)	0.288
ΔHF total	50.85 (148.92)	78.41 (200.61)	0.349
ΔHRV total	63.04 (269.08)	1.11 (560.19)	0.328
ΔLF/HF	−0.07 (2.39)	−4.37 (15.24)	0.081
ΔEKG IBI	−87.16 (77.43)^***^	−84.93 (80.32)^***^	0.342
ΔNN50	−81.15 (84.63)^***^	−52.28 (53.59)^***^	0.020
ΔpNN50	−8.19 (8.55)^***^	−5.33 (5.36)^***^	0.020
ΔSDNN	1.67 (58.33)	26.72 (53.48)^*^	0.116
ΔRMSSD	−0.22 (57.28)	24.89 (51.69)^*^	0.092

*VR, virtual reality; EMG, electromyography; HR/BVP, heart rate/blood vessel pressure; IBI, inter-beat interval; VLF, very-low-frequency band; LF, low-frequency band; HF, high-frequency band; NN50, pNN50, percentage of NN50; number of interval differences of successive normal-to-normal (NN) intervals >50 ms; SDNN, standard deviation of NN; RMSSD, the root mean square of the successive differences*.

a *Subjects who reported a decrease in NRS after a relaxation session from stress phase*.

b *The two groups were compared with the paired t-test when the normality assumption was satisfied and the Wilcoxon signed-rank test if not*.

* *p < 0.05*,

*** *p < 0.001*.

### Changes of Simulator Sickness Questionnaire Scores Before and After VR Application

Table 5 shows the changes of SSQ before and after VR applications during stress phase. Among all subjects, total SSQ score increased significantly from 23.55 (SD = 26.03) to 41.30 (SD = 45.69). In responders, there was no significant difference in SSQ scores at baseline and after VR application. In non-responders, the baseline total score increased from 21.25 (SD = 22.88) to 47.25 (SD = 52.97) after VR application, which was larger than the average of all subjects.

**Table 5:** Changes of SSQ before and after stress exposure using intentionally generated shaking VR.

	**Nausea scores**	**Oculomotor scores**	**Disorientation scores**	**Total scores**
**All subjects (** ***n*** **=** **74)**				
Baseline SSQ	16.50 (22.12)	20.18 (21.27)	27.46 (35.99)	23.55 (26.03)
After VR application during stress phase	32.36 (42.83)	31.86 (31.01)	49.66 (59.73)	41.30 (45.69)
Δ(VR application – baseline)^a^	15.86 (45.31)^**^	11.68 (32.04)^**^	22.20 (57.41)^***^	17.75 (45.64)^***^
**Responders (** ***n*** **=** **39)**				
Baseline SSQ	17.61 (23.01)	22.35 (23.13)	29.62 (41.29)	25.62 (28.70)
After VR application during stress phase	23.97 (35.76)	31.10 (28.19)	43.19 (50.27)	35.96 (37.93)
Δ(VR application – baseline)^a^	6.36 (33.79)	8.75 (27.58)	13.56 (45.33)	10.34 (34.85)
**Non-responders (** ***n*** **=** **35)**				
Baseline SSQ	15.26 (21.36)	17.76 (19.01)	25.06 (29.39)	21.25 (22.88)
After VR application during stress phase	41.70 (49.95)	32.70 (34.28)	56.87 (68.81)	47.25 (52.97)
Δ(VR application – baseline)^a^	26.44 (53.97)^**^	14.94 (36.51)^*^	31.82 (67.82)^**^	26.01 (54.61)^**^

*SSQ, Simulator sickness questionnaire*.

a *Paired t-test*.

* *p < 0.05*,

** *p < 0.01*,

*** *p < 0.001*.

## Discussion

In this study, when VR relaxation or biofeedback was undertaken after stress exposure in people with high stress, although both treatments reduced subjective stress significantly, the difference in subjective stress reduction between the treatments was not significant. When we evaluated the change of stress by means of physiological parameters in both treatments, there was a significant difference in EMG, LF total, LF/HF ratio, and NN50. In particular, the main analysis and sensitivity analysis consistently showed a greater increase of NN50 in VR relaxation than in biofeedback and a greater decrease of EMG in biofeedback than in VR relaxation.

Although, VR was effective in subjective stress reduction in this study, the magnitude of the effect was not significantly different from that of biofeedback. This is consistent with the finding of Rockstroh et al. (44) which compared the effect of relaxation through VR and biofeedback. However, there are differences in methodology between their study and current study. They compared the electrodermal activity and 20-item state version of the STAI between subjects while applying one of four conditions disentangled according to display type (VR vs. computer screen) and biofeedback (electrodermal activity biofeedback vs. no biofeedback) to healthy participants, while we compared the effects of VR relaxation and biofeedback within individuals through a psychological scale and various physiological parameters among highly stressed subjects by crossover design. When evaluated by the subjective scales, the change of STAI-X1, measuring state anxiety, was greater in VR, and the change of NRS, measuring subjective discomfort, was greater in biofeedback. Although, additional research is needed, these findings are presumed to be related to how VR and biofeedback induce relaxation and stress reduction. In the protocol implemented in this study, VR reduced stress by making subjects experience an immersive video showing natural settings that made them feel like they were in a calm and peaceful place, whereas, biofeedback induced relaxation by training participants to consciously affect physiological activities that are generally done unconsciously, by providing feedback on changes in heart rate, respiration rate, or skin conductance.

These differences in scales may be better explained by changes in physiologic parameters. Among the physiological parameters, the decrease in EMG was greater in biofeedback, the increase in NN50 and the decrease in LF/HF ratio were greater in VR, and each change is known to reflect relaxation or stress reduction. The EMG, the sensor of superficial EMG, reflects the degree of muscle tension, which decreases upon relaxation. Therefore, the results of our study suggest that although the feedback of the EMG change was not given to participants according to the protocol, muscle relaxation was done effectively by a conscious control in physiological activities, which was more efficient than by VR relaxation. NN50 was increased more in VR relaxation than by biofeedback, and the result was consistent in both the main analysis and the sensitivity analysis. NN50, one of HRV time-domain measures (69), is closely correlated with peripheral nervous system (PNS) activity (70). Previous studies have shown that when stress is induced, the percentage of NN50 decreases (47, 71). Inferred from these findings, the increase in NN50 in VR relaxation suggests that VR may be more efficient for stress reduction by means of parasympathetic activity. The LF/HF ratio is used to estimate the balance between the sympathetic nervous system (SNS) and PNS, because both PNS and SNS activities contribute to LF power, and PNS activity contributes to HF power (72). A low LF/HF ratio reflects parasympathetic dominance, whereas, a high LF/HF ratio indicates sympathetic dominance (69). Previous studies showed that an increase in the LF/HF ratio is observed in a stress situation (48, 73). Therefore, our finding can be explained by the fact that parasympathetic-dominant physiological responses were induced more by means of VR relaxation, and this leads to reduction of stress.

This study showed the possibility of using VR as a novel tool for stress management. It has been shown that immersion and presence in a natural setting by means of VR have an effect on relaxation, which uses the knowledge that the natural setting generally has a mentally restorative effect (74, 75). VR can be used as a method for providing treatment of new contents as in this study, but it could also be used to effectively deliver existing tools, such as the mindfulness approach or cognitive behavioral therapy (CBT) for management of stress or anxiety. Conventional non-pharmacological interventions have the disadvantage that individual variance is large in motivation, dedication, mindedness, and capacity for conscious control of physiologic responses, all of which affects the outcome of treatment. On the other hand, the effect of individual factors can be alleviated by effectively delivering treatment contents by inducing presence and immersion using visual and auditory stimuli. In addition, the new delivery method by means of VR is more cost-effective than conventional tools, which require well-trained clinicians or therapists. With VR, it is possible to maintain anonymity and facilitate treatment access for users.

Another important finding of this study is that in relaxation through VR, adverse events such as sickness symptoms can interfere with the relaxation process. In this study, we compared the sickness symptoms at baseline and after VR sessions in responders and non-responders. As a result, responders did not show a significant increase in sickness symptoms, whereas, non-responders showed a significant increase in all domains of sickness symptoms. Although, further research is needed, this suggests that there are characteristics of the person receiving benefits from VR relaxation, and along with physiological parameters, this may be an important factor in the selection of methods of non-pharmacological relaxation such as VR or biofeedback.

This study has several limitations. First, exposed stressors in a laboratory environment may not be delivered effectively depending on the individual. In this study, stress exposure was elicited by means of task execution in an intentionally generated shaking VR and cognitive load by means of serial-7 subtraction. We operationally set up this method to elicit stress in a short time in a limited laboratory environment. However, it is not clear whether performing these tasks could be a significant psychosocial stressor for all subjects, because a stressor can be perceived as either challenging or discomforting (76–78). That is, depending on the individual's intrinsic tolerance, the given stress may not be stressful. However, since we targeted people who are expected to have high vulnerability to stress, we expect the discomfort caused by stress exposure to be greater than in the general population. Second, the time to induce stress reduction through VR or biofeedback may not be sufficient. Each relaxation session lasted for a total of 10 min and 30 s, but depending on the individual, the time may be insufficient to induce the maximum level of relaxation. Third, since this study investigated the short-term effect of stress reduction during biofeedback and VR relaxation, further, studies investigating the long-term effect of VR relaxation on stress reduction are needed. Fourth, since this study was conducted with healthy adults, it is difficult to apply the results to children or adolescents. In addition, since the oldest subject was 59 years old, it is difficult to apply our results to the elderly. Fifth, the effect of relaxation through VR or biofeedback should be interpreted in the context of the methodology of study protocol. In this study, e.g., during VR application, the subjects virtually walked without actual movement. The conditions in VR may affect presence and adverse events including cybersickness, and these can also affect relaxation. Regarding biofeedback, in addition to inducing relaxation by feeding back changes in physiological parameters, other methods such as breathing training, progressive muscle relaxation, and guided imagery can be used and the applied protocol may affect the results. In particular, considering that the content of VR relaxation was experiencing a relaxing natural scene, if guided imagery was included in the biofeedback protocol, there might be a difference in the results. Finally, we did not evaluate factors assessing immersion, presence, and interactivity during the VR experience, and these can affect the relaxation through VR.

This study has the following strengths. Although, there was no significant difference in the subjective relaxation between VR and biofeedback, we could identify the physiological changes according to each treatment by comparing changes in various physiological parameters between the treatments within an individual. In addition, by adopting a crossover design, it has the strength of minimizing the confounding effect that could occur due to between-subject variability. This study showed the possibility of VR as a useful tool for stress reduction. In addition to the protocol of VR relaxation applied in this study, future development of new technologies and efficient treatment tools will enable cost-effective, easily accessible, and personalized management of stress.

In conclusion, this study found that VR is effective in reducing subjective stress in people with high stress. Although the effect of VR relaxation was not significantly superior to biofeedback, it showed significant differences in several physiological parameters, and these can act as important factors that affect the selection of non-pharmacological relaxation such as VR in clinical settings.

## Data Availability Statement

The raw data supporting the conclusions of this article will be made available by the authors, without undue reservation.

## Ethics Statement

The studies involving human participants were reviewed and approved by Institutional Review Board of the Samsung Medical Center. The patients/participants provided their written informed consent to participate in this study.

## Author Contributions

HK contributed to interpreting the data and writing the original draft of the manuscript. SK and MJK contributed to statistical analyses. DJK, KK, WHC, and K-AP contributed to data collection and processing. JDKK and DK contributed to production of the virtual reality videos. WHC, K-AP, and HJJ contributed to conceptualization. HJJ contributed to project administration and supervision. All authors contributed to the writing and editing of the manuscript.

## Conflict of Interest

JDKK and DK were employed by Samsung Electronics Co., Ltd. The remaining authors declare that the research was conducted in the absence of any commercial or financial relationships that could be construed as a potential conflict of interest.

## Publisher's Note

All claims expressed in this article are solely those of the authors and do not necessarily represent those of their affiliated organizations, or those of the publisher, the editors and the reviewers. Any product that may be evaluated in this article, or claim that may be made by its manufacturer, is not guaranteed or endorsed by the publisher.
