# Supplementary material for: Effect of Virtual Reality on Stress Reduction and Change of Physiological Parameters Including Heart Rate Variability in People With High Stress: An Open Randomized Crossover Trial
Source: Front Psychiatry. 2021 Aug 10;12:614539. doi: 10.3389/fpsyt.2021.614539 (PMC8384255; doi:10.3389/fpsyt.2021.614539)
Supplement: Supplementary file 1 [file Data_Sheet_1.docx]

**Supplementary Table 1 |** Physiological parameters throughout VR session (n = 74).

|  | Baseline | Stress exposure | VR Relaxation | *p***^a^** |
| --- | --- | --- | --- | --- |
|  | Mean (SD) | | |  |
| EMG | 2.58 (1.99) | 3.93 (4.04) | 3.09 (3.07) | 0.006 |
| Skin Conductance | 0.53 (0.63) | 1.37 (1.22) | 0.94 (0.79) | <0.001. |
| Temperature | 33.10 (1.36) | 31.54 (2.53) | 32.37 (2.40) | <0.001 |
| Respiratory amplitude | 42.38 (6.36) | 42.79 (5.90) | 42.46 (5.66) | 0.453 |
| HR/BVP | 29.69 (0.09) | 29.72 (0.12) | 29.67 (0.11) | <0.001 |
| HR from IBI | 71.52 (11.76) | 72.66 (9.18) | 66.05 (8.12) | <0.001 |
| VLF total | 64.15 (47.09) | 82.55 (61.23) | 85.41 (63.83) | 0.726 |
| LF total | 94.62 (80.12) | 160.78 (95.74) | 143.91 (115.89) | 0.017 |
| HF total | 84.96 (86.89) | 178.50 (167.72) | 109.77 (112.93) | 0.005 |
| HRV total | 255.95 (195.23) | 464.99 (336.16) | 351.74 (239.88) | 0.008 |
| LF/HF | 2.26 (2.27) | 3.03 (2.10) | 2.71 (2.43) | 0.080 |
| EKG IBI | 830.13 (137.44) | 844.46 (109.38) | 924.83 (118.46) | <0.001 |
| NN50 | 70.26 (80.39) | 26.30 (21.06) | 96.11 (90.91) | <0.001 |
| pNN50 | 8.24 (9.09) | 3.08 (2.51) | 10.17 (9.57) | <0.001 |
| SDNN | 52.57 (36.87) | 60.17 (59.88) | 43.34 (55.19) | 0.128 |
| RMSSD | 61.36 (38.87) | 71.81 (60.71) | 56.72 (59.07) | 0.184 |

*VR,* virtual reality; *EMG,* electromyography; *HR/BVP,* heart rate/blood vessel pressure; *IBI,* inter-beat interval; *VLF,* very low frequency band; *LF,* low frequency band; *HF,* high frequency band; *NN50,* number of interval differences of successive normal-to-normal (NN) intervals greater than 50 ms; *pNN50*, percentage of NN50; *SDNN,* standard deviation of NN; *RMSSD,* the root mean square of the successive differences.

**^a^**The values after stress exposure and relaxation were compared with the paired *t*-test when the normality assumption was satisfied and the Wilcoxon signed-rank test if not.

**Supplementary Table 2 |** Changes of physiological parameters throughout biofeedback session (n = 74).

|  | Baseline | Stress exposure | Biofeedback  Relaxation | *p***^a^** |
| --- | --- | --- | --- | --- |
|  | Mean (SD) | | |  |
| EMG | 2.60 (2.83) | 6.81 (20.72) | 2.40 (2.94) | <0.001 |
| Skin Conductance | 0.52 (0.53) | 1.37 (1.32) | 0.86 (0.74) | <0.001 |
| Temperature | 33.22 (1.31) | 31.96 (1.97) | 32.75 (1.50) | <0.001 |
| Respiratory amplitude | 42.49 (6.19) | 42.04 (6.20) | 42.19 (5.73) | 0.955 |
| HR/BVP | 29.69 (0.08) | 29.79 (0.57) | 29.72 (0.49) | <0.001 |
| HR from IBI | 72.93 (9.43) | 73.21 (9.84) | 67.56 (8.28) | <0.001 |
| VLF total | 67.69 (52.04) | 77.31 (61.88) | 97.91 (73.23) | 0.027 |
| LF total | 88.94 (82.80) | 175.42 (184.72) | 213.99 (267.73) | 0.489 |
| HF total | 69.63 (71.88) | 149.82 (227.64) | 85.34 (68.00) | 0.217 |
| HRV total | 235.86 (190.52) | 440.43 (504.94) | 407.01 (333.58) | 0.440 |
| LF/HF | 2.20 (2.08) | 3.28 (2.41) | 5.94 (12.30) | 0.527 |
| EKG IBI | 836.71 (114.89) | 832.22 (103.80) | 906.68 (118.67) | <0.001 |
| NN50 | 62.87 (65.84) | 21.10 (18.12) | 71.84 (65.57) | <0.001 |
| SDNN | 49.17 (46.67) | 56.94 (56.61) | 35.53 (33.62) | 0.005 |
| RMSSD | 57.92 (52.10) | 67.79 (59.90) | 46.84 (34.31) | 0.008 |

*EMG,* electromyography; *HR/BVP,* heart rate/blood vessel pressure; *IBI,* inter-beat interval; *VLF,* very low frequency band; *LF,* low frequency band; *HF,* high frequency band; *NN50,* number of interval differences of successive normal-to-normal (NN) intervals greater than 50 ms; *SDNN,* standard deviation of NN; *RMSSD,* the root mean square of the successive differences.

**^a^**The values after stress exposure and relaxation session were compared with the paired *t*-test when the normality assumption was satisfied and the Wilcoxon signed-rank test if not.

**Supplementary Figure 1 |** Changes of STAI-X-1 from stress exposure to relaxation session according to the type of relaxation session


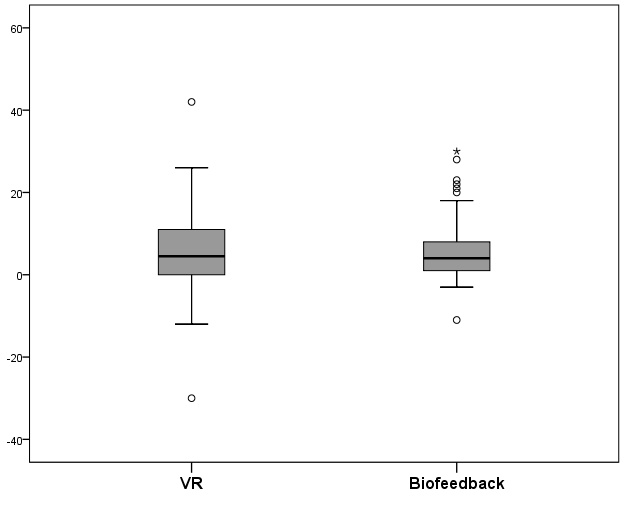


*STAI,* State-Trait Anxiety Inventory

**Supplementary Figure 2 |** Changes of NRS from stress exposure to relaxation session according to the type of relaxation session

*
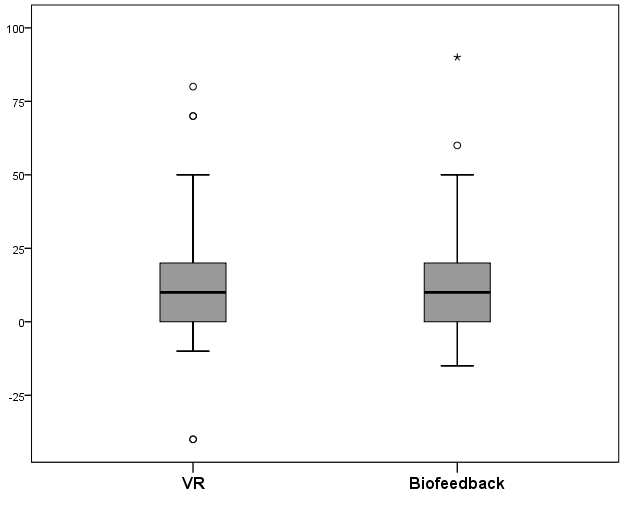
*

*NRS,* Numeric Rating Scale
